# The causal explanatory functions of medical diagnoses

**DOI:** 10.1007/s11017-016-9377-5

**Published:** 2016-09-16

**Authors:** Hane Htut Maung

**Affiliations:** Department of Politics, Philosophy, and Religion, Lancaster University, Lancaster, LA1 4YL UK

**Keywords:** Diagnosis, Explanation, Causation, Mechanisms

## Abstract

Diagnoses in medicine are often taken to serve as explanations of patients’ symptoms and signs. This article examines how they do so. I begin by arguing that although some instances of diagnostic explanation can be formulated as covering law arguments, they are explanatory neither in virtue of their argumentative structures nor in virtue of general regularities between diagnoses and clinical presentations. I then consider the theory that medical diagnoses explain symptoms and signs by identifying their actual causes in particular cases. While I take this to be largely correct, I argue that for a diagnosis to function as a satisfactory causal explanation of a patient’s symptoms and signs, it also needs to be supplemented by understanding the mechanisms by which the identified cause produces the symptoms and signs. This mechanistic understanding comes not from the diagnosis itself, but rather from the theoretical framework within which the physician operates.

## Introduction

The clinical encounter between patient and physician usually begins with the physician taking a history from the patient to elicit information about his or her symptoms and other relevant concerns, examining the patient to observe any signs, and reviewing any available test results [1, 2]. After gathering and consolidating the information about symptoms, signs, and test results (henceforth referred to collectively as ‘patient data’), the physician infers a diagnosis. For example, a patient may present with shortness of breath, leg oedema, and an abnormal electrocardiogram, from which the physician infers the diagnosis of heart failure.

In turn, the diagnostic hypothesis is normally taken to be an explanation of the patient data [3–8]. Tomasz Rzepiński characterises a diagnosis as an answer to the following kind of contrastive question about the patient: ‘“Why X1, X2, …, Xn, when it should be Y1, Y2, …, Yn?” where X1, X2, …, Xn account for a description of improper symptoms, while Y1, Y2, …, Yn account for a description of a properly functioning human body’ [1, p. 70]. For instance, the diagnosis of heart failure explains why the patient has shortness of breath, leg oedema, and an abnormal electrocardiogram, rather than having normal breathing, the absence of oedema, and normal sinus rhythm. Hence, there is a bidirectional relation between patient data and diagnosis: the diagnosis is inferred from the patient data, and the patient data is explained by the diagnosis.

This explanatory function of a diagnosis is desirable for a number of purposes. First, it can support predictions about likely future outcomes. For example, knowing that the patient’s shortness of breath is due to heart failure supports the prediction that the condition is likely to have a chronic course and involve other symptoms, such as leg oedema. Second, where treatment is available, an explanation can inform decisions regarding therapeutic interventions. Caroline Whitbeck argues that the purpose of a diagnosis is to reveal ‘the *causes* and *mechanisms* of a patient’s disease insofar as this information is needed to inform treatment and management decisions to achieve the best medical outcome for the patient’ [9, p. 324]. By explaining that the patient is short of breath because of heart failure, the diagnosis provides the physician with knowledge that justifies the decision to administer targeted treatments for heart failure to reverse the patient’s shortness of breath. Third, some theorists note that the explanation provided by a diagnosis is of value to the patient because it legitimises illness [7, 10] and can offer a sense of relief [11, 12].

The aim of this article is to provide an account that characterises how a diagnosis explains patient data. I argue that traditional covering law models of explanation fail to adequately capture this explanatory relation. Instead, I propose that where a diagnosis successfully explains patient data, it does so (1) by identifying the cause of the patient data and (2) in the presence of theoretical understanding of the mechanisms that link the identified cause to the patient data. My approach in this article is predominantly descriptive, but has normative implications. On the descriptive side, the model of explanation I provide is intended to capture, with fidelity, the nature of explanation in paradigm cases where diagnoses explain the patient data. However, in the course of my discussion, I show that some of the traditional models of explanation fail to capture how diagnoses explain patient data on the grounds that they permit spurious or incorrect diagnoses, which has normative implications for how physicians should and should not reason.

Although a substantial amount of literature has been dedicated to the logic of diagnostic reasoning in medicine [2, 13–17] and the nature of explanation in the biomedical sciences [4, 5, 18], this particular topic of how diagnoses serve as explanations of patient data has been underexplored in the philosophy of medicine. The literature on diagnostic reasoning has largely focused on analysing the inferential process leading from the patient data to the diagnostic hypothesis [2, 13–17], but little has been written about the nature of the explanatory relation that goes in the opposite direction, from the diagnosis to the patient data. Kazem Sadegh-Zadeh, for example, provides a thorough analysis of diagnostic reasoning based on probabilistic causal analysis and fuzzy logic, and while he does state that it is ‘usually required that the diagnosis *causally explain* the patient data’ [16, p. 329], the analysis he provides is more an account of the logical process of generating a diagnostic hypothesis from the patient data, rather than of how a diagnosis serves as an explanation of the patient data [16, pp. 598–603]. The same can also be said of the literature on artificial intelligence and expert diagnostic systems, which looks at the development of statistical algorithms to simulate and enhance clinical decision-making [3, 16, 19]. Again, the focus of this literature is the process of analysing patient data to arrive at a diagnosis. While the outcome of this process may be an explanation of the patient data, an account of precisely what makes it explanatory is still wanting. Hence, the account of explanation I provide can be seen as complementing, rather than challenging, the above mentioned work on diagnostic reasoning.

The philosophical literature on the nature of explanation in the biomedical sciences has largely focused on explanation in the context of medical research, rather than explanation in the context of clinical practice [4, 5, 18]. Nonetheless, there are notable exceptions of particular relevance to my discussion. One is Kenneth Schaffner, who argues that explanation in the biomedical sciences is different from that in the physical sciences because the former involves qualitative and analogical reasoning from loose theoretical generalisations rather than the subsumption under laws that is involved in the latter [18, 20]. In his 1986 paper [20], Schaffner shows how such analogical reasoning is used to understand individual cases in medicine. However, his is a general account of how theoretical knowledge of medical science is applied to individual cases, not a specific analysis of how diagnoses in particular serve as explanations of patient data. While he notes that a physician’s theoretical knowledge consists of ‘a repository of classificatory or nosological generalizations’ and ‘a grounding in the basic sciences of biochemistry, histology, physiology, and the pathological variants of the “normal” and “healthy” processes’ [20, p. 71], his general account runs these elements together. As such, it is not made explicitly clear how this extension of theoretical knowledge exactly relates to the specific epistemic role of a diagnosis in the clinical encounter. One of my contributions in this article, then, will be to show precisely where and how Schaffner’s insightful account of analogical reasoning from theoretical generalisations applies to the explanatory function served by a diagnosis.

Another theorist relevant to my discussion is Margherita Benzi, who, in a recent paper [21], makes the important step of specifically and explicitly applying philosophical theory on causal explanation to the topic of diagnosis. Benzi proposes that diagnoses are not explanatory in virtue of general causal regularities between them and the patient data but rather because they pick out the actual causes of the patient data in each individual case. While I take this to be correct, I argue that it is incomplete as it stands and needs to be supplemented with consideration of how the intelligibility of the explanation also rests on knowledge of the mechanisms linking the identified cause and the patient data. I show that it is here that we can appreciate the relevance to diagnostic explanation of Schaffner’s insights, which Benzi briefly mentions in her paper [21, p. 368] but does not integrate into her account.

The rest of the article is structured as follows. In the second section that follows, I argue that some of the traditional models of explanation in the philosophy of science fail to adequately capture the explanatory relation between diagnosis and patient data. In particular, I consider Carl Gustav Hempel’s deductive-nomological and inductive-statistical models of scientific explanation [22], and I argue that diagnostic explanations are not explanatory in virtue of either their argumentative structures or the general regularities between the diagnoses and the patient data. In the third section, I present Benzi’s argument that medical diagnoses explain by identifying the actual causes of the patient data in individual cases rather than by subsuming them under general causal regularities [21]. I then argue in the fourth section that although Benzi is correct to stress that diagnostic explanation appeals to actual causation, a more complete account also needs to consider how a successful causal explanation of a patient’s symptom presentation not only involves a simple causal claim of the form ‘*C* causes *E*’, but also relies on mechanistic causal knowledge of the form ‘this mechanism produces this phenomenon’ [23, p. 20]. In the fifth section, I suggest that the former is the outcome of the diagnostic search, while the latter is provided by the theoretical framework in which the physician operates. This is supported with appeal to Schaffner’s work on theoretical generalisations in medicine [20], as well as Jeremy Simon’s recent work on disease ontology [24].

Before I proceed, I offer two clarifications. The first is a note on terminology. As noted by Mildred Blaxter, ‘diagnosis’ is an ambiguous term that can refer to either a category or a process [25]. A clinician may use the term to denote the condition from which the patient is suffering, such as ‘the diagnosis is acute appendicitis’, or to indicate the process by which this conclusion can be reached, such as ‘the diagnosis is clinical and radiological’. To avoid this ambiguity, I reserve the term ‘diagnosis’ to refer to the categorical conclusion and refer to the process leading to the conclusion as the ‘diagnostic process’. Second, I do not claim that all diagnoses function as explanations of symptoms and signs. In some cases, the diagnoses may be syndromic. That is to say, they do not refer to underlying diseases, but to the constellations of symptoms and signs themselves. Examples include some diagnoses in psychiatry and some of the so-called medically unexplained syndromes, whose explanatory statuses are hotly debated [7, 8, 12]. Hence, my aim, more accurately, is to show how diagnoses explain patients’ symptoms and signs only in the cases where they explain them at all.

## Covering law models

Among the most influential and widely discussed accounts of scientific explanation in the philosophical literature is Hempel’s covering law account, according to which a phenomenon is explained by subsuming it under a general law or regularity [22]. A covering law explanation has the form of an argument, whereby the *explanandum* is concluded from a set of premises, of which at least one must be a general law that is necessary for the argument. The argument can be either deductive or inductive. The former kind, known as deductive-nomological explanation, has the following form when applied to diagnostic explanation, where *S* is a set of patient data, *D* is the diagnosis, and *D* → *S* is the general law linking the diagnosis with the set of patient data:
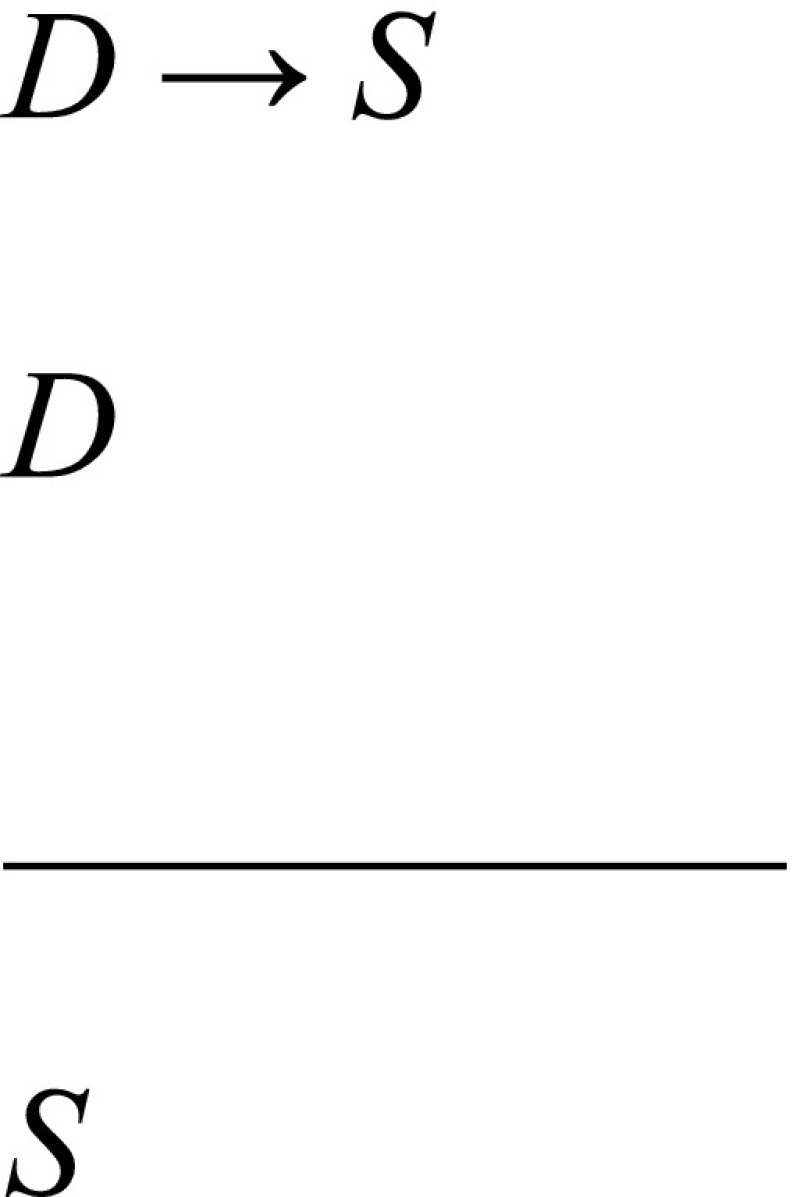
 For instance, according to the deductive-nomological model, a patient’s leg oedema would be explained by deducing it from the diagnosis of heart failure and the general law that links heart failure with leg oedema.

At first glance, it might appear that this is getting things backwards, as physicians do not typically begin with a diagnosis and deduce the patient data, but begin with the patient data and then infer a diagnosis as an explanation. However, this problem is only apparent and disappears with a more accurate understanding of what the deductive-nomological formulation is intended to capture. The deductive-nomological formulation is not intended to be a historical representation that captures the psychological process of hypothesis formation, but rather an atemporal representation that captures the logical relation between the hypothesis and the data. Under the model, then, the explanatory relation between the *explanans* and the *explanandum* does not depend on how we arrived at the former, but on whether the latter can be deductively entailed from the former.

Nonetheless, the deductive-nomological model has a serious limitation in the context of clinical practice. Many regularities in medicine are probabilistic rather than deterministic and so do not enable sound deductions of the patient data from the diagnoses [16, p. 344]. In the above mentioned example, the correlation between heart failure and leg oedema is not absolute, and it is possible to have heart failure without leg oedema. This suggests that the premise *D* → *S* is false and that the deduction is not sound. Therefore, the deductive-nomological model is only applicable to a very limited number of cases of diagnostic explanation.

Hempel concedes that the deductive-nomological model cannot account for cases of explanation that do not involve deterministic laws and introduces the latter kind of covering law argument, known as inductive-statistical explanation, to make up for these cases. According to this, to explain a phenomenon is to inductively infer it from a statistical generalisation about previously observed cases. Hempel uses the example of Jones’ recovery from a streptococcal infection explained by his having taken penicillin and the statistical generalisation that a high proportion of people who have streptococcal infections recover after taking penicillin. Applied to the example of heart failure, the patient’s leg oedema is explained by the fact that he or she has heart failure, along with the statistical generalisation that a high proportion of people of patients with heart failure have leg oedema:
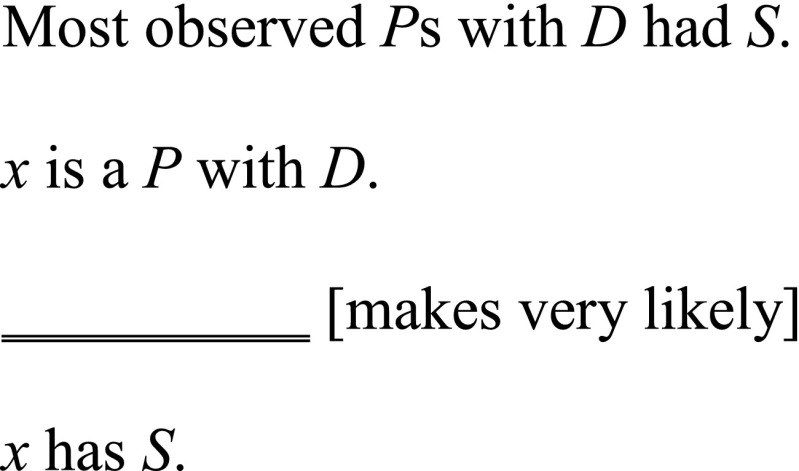
 The inductive-statistical model accommodates the fact that many relations between diagnosis and symptoms in medicine are probabilistic [4, p. 203]. Therefore, a charitable rendering of a covering law account of diagnostic explanation needs to allow inductive-statistical as well as deductive-nomological explanations.

I accept that some instances of diagnostic explanation may be formulated as covering law arguments of the inductive-statistical kind. There is a certain feature of diagnoses that permits such a formulation. Covering law explanations appeal to laws or regularities, which in turn depend on the presupposition of repeatable types that instantiate these laws or regularities. In medicine, diagnoses are often treated as such repeatable types [16, pp. 155–156]. They are generalised categories, whose tokens are taken to share certain properties. For example, heart failure is considered to be a type characterised by the following: ‘heart failure is the state of any heart disease in which, despite adequate ventricular filling, the heart’s output is decreased or in which the heart is unable to pump blood at a rate adequate for satisfying the requirements of the tissues with function parameters remaining within normal limits’ [26, p. 445]. Individual cases of heart failure are tokens of this type that instantiate this feature. This characterisation of diagnoses as repeatable types enables them to support the kinds of regularity and inductive inference that feature in inductive-statistical explanations.

However, it has long been argued that the inductive-statistical model as it stands is too permissive to be a complete account of explanation. There are well-known counterexamples that fulfil the requirements of the inductive-statistical model yet are not genuinely explanatory. One kind of counterexample concerns explanatory irrelevancies. Peter Achinstein gives the hypothetical case of Jones, who dies within a day of eating a pound of arsenic [27]. Assume that the actual cause of Jones’ death had been an unrelated car accident. If this is the case, then his eating a pound of arsenic is explanatorily irrelevant to his dying. However, according to the inductive-statistical model, Jones’ death would still be explained by his eating a pound of arsenic, along with the statistical generalisation that a very large proportion of people who eat a pound of arsenic die within a day. To take another example, a significant proportion of patients diagnosed with left hemispheric stroke present with right-sided paralysis. Now, consider the case of a patient diagnosed with left hemispheric stroke but who already has right-sided paralysis for a different reason, such as cerebral palsy. In this case, the diagnosis of left hemispheric stroke is explanatorily irrelevant to the patient’s right-sided paralysis. Nonetheless, according to the covering law account, the patient’s right-sided paralysis would still be explained by his or her diagnosis of left hemispheric stroke, along with the statistical generalisation that a large proportion of patients diagnosed with left hemispheric stroke present with right-sided paralysis.

Another kind of counterexample concerns spurious correlations. Wesley Salmon gives the example of a correlation between a falling barometer reading and a storm [28]. Although there is a significant statistical regularity between these two event types, a falling barometer reading is not a legitimate explanation of a storm. Rather, both have a common explanation, namely, the preceding drop in atmospheric pressure. Applying this to a medical example, there is a statistical regularity between calf pain and pulmonary embolism, such that the probability of a patient having calf pain is higher if he or she also has a pulmonary embolism than the probability of his or her having calf pain under any circumstance. However, in this case, the diagnosis of a pulmonary embolism does not explain the patient’s calf pain. Rather, both the calf pain and the pulmonary embolism, as well as the statistical relation between the two, can be explained by the diagnosis of deep vein thrombosis.

The above counterexamples show that genuine explanatory relations are underdetermined by covering law arguments. In the example of the patient with right-sided paralysis, there are two possible explanations for the patient data, each supported by a different inductive-statistical argument. These are left hemispheric stroke and cerebral palsy, respectively. Here, the correct explanation cannot be determined by the inductive-statistical model on its own. Rather, confronted with two inductive-statistical arguments supporting different diagnoses, the physician has to make a choice, or an inference to the best explanation, based on some other criterion. Hence, the covering law account at best describes only a part of the relation between the actual diagnosis and the clinical data.

What seems to be suggested by the above counterexamples is that the necessary criterion for the relation between the diagnosis and the patient data to be genuinely explanatory is causation. In the case of the patient with cerebral palsy, the reason why left hemispheric stroke does not explain his or her right-sided paralysis is that the right-sided paralysis was caused by another condition, namely, cerebral palsy. Also, in the case of the patient with deep vein thrombosis and pulmonary embolism, the reason why the former but not the latter explains his or her chest pain is because it is the former that had caused it. However, inductive-statistical relations are not specifically causal, so on their own, they cannot distinguish between diagnoses that genuinely explain the patient data and those that are merely correlated with the patient data. The upshot, then, is that while the covering law account as described above may capture a part of the relation between a diagnosis and the patient data, it fails to pick out specifically what it is that makes this relation genuinely explanatory.

## Causal explanation and actual causation

The above considerations suggest that an adequate model of diagnostic explanation must take causation into account. Over the past half century, the causal model of explanation has attracted a large number of proponents in the field of philosophy of science [28–32]. The basic claim of the causal model is that to explain something is to provide information about its cause. This certainly has intuitive appeal with respect to diagnostic explanation as it is commonly suggested that the aim of the diagnostic process is to search for the cause of the clinical manifestation [6, 9, 15]. Furthermore, the model’s requirement of a causal connection between the *explanandum* and the *explanans* helps to avoid the over-permissiveness of the covering law account. As noted in the previous section, physicians seeking explanations of patient data may be confronted with various factors that are correlated with the patient data, some of which may be causally irrelevant or spurious but nonetheless may satisfy the requirements for inductive-statistical explanations. Under the causal model of explanation, though, only those correlations which are genuinely causal would qualify as being explanatory.

Although the causal model of explanation is sometimes described as a reaction to the covering law account, some instances of causal explanation can be formulated as special cases of covering law explanation where the regularities appealed to are causal regularities, or ‘laws of succession’ [22, p. 352]. Physics and chemistry contain such examples. For example, one could explain why the ice cube in a glass of water melts by appealing more generally to the laws describing how high temperatures influence the hydrogen bonds between H_2_O molecules. As noted in the section above, some instances of diagnostic explanation can be formulated as covering law arguments, which suggests that they could be considered cases of covering law explanation that appeal to causal, rather than merely statistical, regularities.

Benzi notes that in covering law explanations, the causal regularities hold between general types [21, p. 367]. I have already shown in the previous section how a diagnosis, such as heart failure, is treated as a repeatable type. The covering law account also treats the symptom presentation as a repeatable type, such that a causal regularity is taken to hold between the type diagnosis ‘heart failure’ and the type symptom ‘leg oedema’. In diagnostic explanation, however, the *explanandum* is not a generality, but a particular fact. That is to say, in the case where the diagnosis of heart failure successfully explains leg oedema, what is being explained is not why leg oedema occurs in general at the total population level but rather why this particular patient has leg oedema. To particularise the general regularity to the individual case, the covering law account treats the individual case as a token of the general type to which the regularity applies. According to this approach, the individual case of leg oedema is explained by the diagnosis of heart failure because it is a token of the type ‘leg oedema’ and there is a causal regularity between the type ‘heart failure’ and the type ‘leg oedema’.

Indeed, in many cases, the explanation of the individual case as if it is a token of a homogeneous type would turn out to yield the correct diagnosis. If a particular type of condition is statistically the commonest cause of a type of symptom in the total population, then it follows that most individual cases of this symptom would be caused by this condition. However, Benzi argues that this does not capture all cases of diagnostic explanation [21, pp. 367–368]. She draws on Samuel Gorovitz and Alasdair MacIntyre’s observation that what is crucially important about individual cases in medicine is what is distinctive about them as particulars (see [33]). Far from being tokens of a homogeneous type, the particular cases of a certain clinical presentation are affected by so many contingencies as to make each case unique. Given this uniqueness, the general causal regularity appealed to in a covering law argument may fail to pick out the actual causal relation in a given case. In other words, the likeliest cause of a clinical presentation in the relevant reference class may not be the actual cause of the clinical presentation in a particular patient.

Consider Benzi’s example of a patient presenting to primary care with a new onset of leg oedema, which in this particular case turns out to be caused by acute kidney disease [21, p. 369]. Also consider that this patient is known to already have a longstanding history of heart failure. Under the covering law account, the leg oedema could be explained with appeal to a causal regularity between kidney disease and leg oedema. However, in the primary care population, leg oedema is more likely to be caused by heart failure than by kidney disease [34]. Hence, the causal regularity between heart failure and leg oedema would also satisfy the requirements of a covering law explanation, despite this not being the actual cause of the leg oedema for this particular patient. The upshot is that appealing to general causal regularities cannot discern the actual explanation from the spurious one in the particular case, and so fails to capture what it is that makes the relation between a diagnosis and a set of patient data genuinely explanatory.

Benzi’s solution, then, is to propose that the relation between a diagnosis and the patient data is explanatory not in virtue of a general causal regularity but in virtue of the actual cause of the patient data in the given case [21]. That is to say, a diagnosis explains the patient data if it identifies the actual cause of that patient data. Hence, in the above mentioned example, heart failure may be a more common cause of leg oedema than kidney disease in the general population, but the correct explanation of leg oedema in the given patient is kidney disease, not heart failure, because kidney disease is the actual cause of the leg oedema in that particular case.

The proponent of the covering law account might respond by suggesting that the relevant reference class to which the general causal regularity applies could be narrowed down by including the details of the contingencies emphasised by Gorovitz and MacIntyre [33] in the description of the reference class. For example, the description of the relevant reference class would not simply be ‘leg oedema’, but something like ‘leg oedema, male, elderly, smoker, hypertensive, diabetic, proteinuria, raised serum creatinine, family history of kidney disease…’, which would strengthen the statistical relation between the reference class to which the patient belongs and the diagnosis of kidney disease. However, there are two problems with this suggestion. First, as argued by Nancy Cartwright [35] and restated by Stefan Dragulinescu [36], a complete description that achieves absolute concordance between the reference class and the correct diagnosis may not be possible. Although we can include certain known risk factors in the description of a reference class, there are also many other contingencies for which we cannot account due to our ignorance of them [33, p. 16]. To paraphrase Cartwright, there may be no available complete description, but simply individual variation [35]. Second, even if, *à la* Laplace’s demon, we were able to specify all of the relevant contingencies and include them in a description, the sheer number of contingencies required to achieve absolute concordance between a reference class and a diagnosis would make the reference class so narrow that we can no longer claim that what we are appealing to in diagnostic explanations are ‘general causal regularities’ rather than instances of singular causation.

So, an adequate causal account of diagnostic explanation cannot be based on general causal regularities, but it needs to appeal to the notion of actual causation in each individual case. As argued by Benzi, the *explanandum*, or the patient’s clinical presentation, cannot be characterised as a token of a type, but as a distinctive particular [21, pp. 367–368]. The *explanans*, or the diagnosis, explains by identifying the actual cause of the clinical presentation in the particular patient. This not only marks an ontological shift from Hempel’s covering law account due to the commitment to actual causal connections rather than regularities, but it also offers an epistemic shift due to its departure from the claim that explanations are necessarily arguments.

What has been presented here is a descriptive account of what constitutes the explanatory relation between a diagnosis and the patient data, but it does have normative implications for how physicians should reason. It supports the idea, suggested by Dominick Rizzi [15, p. 316], that while appeal to causal regularities is of relevance to the scientific understanding of what causes a condition in general, it is singular causation that is relevant to the diagnostic process, where the goal is to ascertain the cause in the individual case. The importance of this is that one of the key functions of a diagnosis is to help determine the correct intervention for the given patient. Settling for the diagnosis of heart failure as an explanation of leg oedema on the grounds that it is normally the cause of leg oedema in general could have disastrous consequences for the patient whose leg oedema is actually caused by a different condition. Of course, it may be that the precise identification of the actual cause in a given case is not immediately possible due to limitations of resources in the given setting, in which case the best that the physician can practically do may be to treat the patient as a token of a type and infer a most likely cause based on knowledge of causal regularities. I do not dispute that such reasoning may be justified; indeed, it may likely be successful given the context. However, with respect to the epistemic status of the resulting relation between the conjectured diagnosis and the patient data, Benzi’s analysis suggests that this relation would only be genuinely explanatory if the inferred likeliest cause does indeed match the actual cause of the patient data in the given case. A diagnosis that cites the wrong cause of the patient data cannot be said to explain the patient data.

## Causes and mechanisms

Benzi is correct to characterise medical diagnoses as causal explanations of symptoms based on particulars [21]. In clinical practice, the diagnostic process is normally aimed at discovering the pathology that is causing a particular patient’s symptoms and signs. The diagnosis, which is the outcome of this process, often denotes this cause. For example, the diagnosis of acute appendicitis points to inflammation of the appendix as the cause of a patient’s abdominal pain, and the diagnosis of myocardial infarction points to ischaemic necrosis of the heart muscle as the cause of a patient’s chest pain.

The above suggests that a diagnostic explanation assumes the form of a simple causal claim, ‘*C* causes *E*’, where *C* is the pathology picked out by the diagnosis and *E* is the patient data in need of explanation. This conforms to the account of causal explanation advocated by David Lewis, according to whom ‘an explainer might give information about the causal history of the *explanandum* by saying that a certain particular event is included therein’ [30, p. 219]. Benzi appears to assume this approach in some passages, such as her counterfactual analysis of a heart problem and a kidney problem as potential causes of a patient’s leg oedema [21, pp. 369–370].

While I agree with this characterisation of a diagnosis as identifying *C* as the cause of *E*, I argue that its explanatory strength depends on an understanding of how *C* produces *E*. In other words, knowledge of the causative pathology needs to be supplemented with some knowledge of the mechanisms by which this pathology causes the symptoms. For example, the diagnosis of heart failure may point to the failure of the heart to pump sufficiently as the cause of the patient’s leg oedema, but this is of limited explanatory value unless it is accompanied by knowledge of the mechanisms by which this failure of the heart to pump sufficiently produces the leg oedema. While Benzi does briefly mention mechanisms in her discussion, it is not made clear how they fit into the account of causal explanation presented [21, p. 366].

The role of mechanisms in explanation has recently received a lot of attention from philosophers of science. This is, to some degree, inspired by Salmon’s mechanistic conception of causation [37], which contrasts with the counterfactual conception of causation advocated by Lewis [30]. However, as noted by Viorel Pâslaru [38], more recent philosophers are in disagreement over how the precise nature of a mechanism should be understood. Some authors take causes to be reducible to mechanisms. For example, Stuart Glennan [39] argues that causal relations can be explained by mechanisms, while Peter Machamer, Lindley Darden, and Carl Craver [40] suggest that the concept of ‘cause’ is vague and can be replaced with more precise mechanistic concepts such as ‘push’, ‘carry’, ‘burn’, and so on. By contrast, James Woodward suggests that mechanisms are reducible to causes and can be analysed counterfactually [41]. Nonetheless, despite these metaphysical disagreements, it is generally agreed that a mechanistic explanation for a phenomenon should include mention of (1) component parts and (2) their activities organised in such a way that they produce the phenomenon. This is sufficient for my present analysis of medical explanation, so the metaphysical debate regarding whether mechanisms can be reduced to causes or *vice versa* can be set aside.

The mechanistic conception of causal explanation has had considerable success in the philosophy of medicine with respect to analyses of disease causation. Examples include Lindley Darden’s [23] discussion of the genetic basis of cystic fibrosis, Federica Russo and Jon Williamson’s [42] analysis of the relation between smoking and bronchial carcinoma, and Mauro Nervi’s [43] analysis of pathological processes. Theorists such as those mentioned above argue that explanations that appeal to mechanisms are desirable in the biomedical sciences because they provide more detail than simple causal claims, offer justification for believing that a correlation is genuinely causal, inform predictions about outcomes, and identify targets for intervention.

I argue that these also apply to the explanation of patient data in the clinical context. Knowledge of mechanisms makes the causal connection between a diagnosis and the patient data more intelligible. This is perhaps most obvious in the case where a pathological process located in one organ system produces symptoms and signs located in seemingly unrelated organ systems. For example, consider the case of a patient who presents with the recent onset of abdominal obesity, muscle weakness, and fragile skin, and is diagnosed with lung carcinoma. This may correctly identify the cause of the patient data, but it is of limited explanatory value on its own due to the apparent gap between cause and effect. However, the connection is more intelligible if it is known that a small cell lung tumour can secrete adrenocorticotropic hormone, which stimulates the adrenal glands to secrete cortisol, which in turn alters lipid and protein metabolism. Here, the presence of a plausible mechanistic story linking *C* and *E* provides justificatory support for the claim that *C* is the cause of *E*, thus substantiating the value of invoking *C* as a causal explanation of *E*.

Another reason this mechanistic knowledge is important is that it supports the prognostic and therapeutic aims of medicine. Holly Andersen argues that knowledge of mechanisms can ‘provide grounds for prediction about what would happen to a phenomenon of interest given specific interventions on it’ [44, p. 993]. While identifying *C* as the cause of *E* may suggest that treatment ought to intervene on *C* or on somewhere along the causal chain from *C* to *E*, knowing the mechanisms by which *C* produces *E* allows us to isolate particular targets for intervention and, moreover, gives us an indication of how to intervene on these targets. This squares with the notion that the causal information required in an explanation is relative to our explanatory interests, which in clinical medicine are largely to inform prognosis and guide treatment and prevention. Whitbeck, for example, argues that the diagnostic process aims for ‘whatever degree of identification is necessary to achieve the best outcome for the patient and to prevent the spread of disease’ [9, p. 322]. For this purpose, it may not be enough merely to identify *C* as the cause of *E*, but we may also need to know further details of how *C* produces *E*. Conversely, the prognostic and therapeutic aims of medicine impose negative constraints on how much mechanistic detail is considered relevant in a causal explanation. As Nervi notes, refining a mechanistic account too much may yield ‘elementary biochemical events of little or no interest to the researcher’ [43, p. 227]. Hence, details that do not aid prediction or intervention in any relevant way may be considered superfluous to the explanation.

The above considerations highlight the importance of mechanistic knowledge in the clinical context of diagnosis. While Benzi is correct that the contribution of the diagnosis is to identify the actual cause of the patient data [21], further knowledge of the mechanisms linking this identified cause and the patient data is usually needed for this to be of explanatory value. In the following section, I examine more closely the sources of this mechanistic knowledge.

## Mechanisms in a theoretical framework

So far, I have argued that a diagnosis explains patient data *E* by identifying pathology *C* as its cause, but the explanatory value of ‘*C* causes *E*’ also depends on understanding the mechanisms by which *C* produces *E*. This raises the question of whence this mechanistic knowledge comes. The account of actual causation presented in the third section above would suggest that the mechanistic knowledge is not explicitly contained in the diagnosis itself, which identifies and denotes the causative pathology *C*. For example, the diagnosis of heart failure explicitly refers to the failure of the heart to pump sufficiently to meet the body’s metabolic requirements, but this description by itself does not provide information about the mechanisms by which leg oedema is produced. Therefore, in such a case, the knowledge of mechanisms must come from sources beyond what is explicitly contained in the diagnosis itself. I suggest that it comes from the broader theoretical framework in which the physician operates.

Simon presents a way of thinking about disease ontology that fits well with this idea. He argues that a model of a disease consists of an explicit description and an implicit addition. The explicit description is the specification of the intrinsic structure of an essential pathological feature. The implicit addition is relational, namely, the assumption that this pathological feature is ‘embedded in an otherwise unspecified living human being, or, more precisely, in an abstract system representing the general physiological features of a living human being’ [24, p. 360]. For instance, he suggests that cystic fibrosis is defined, in essence, by an abnormal CFTR ion transport system, but there is an implicit assumption that this abnormal CFTR ion transport system occurs within and influences a broader physiological system. As noted by Simon, “[a] cell cannot have cystic fibrosis by itself” [24, p. 364]. Although Simon’s account is presented as a metaphysical analysis of the ontological structures of diseases rather than an account of causal explanation in medicine, it does have an important epistemic implication, namely, that knowledge of diseases is embedded within a broader theoretical framework of pathophysiological principles.

A useful way to think about the structure of this theoretical framework is provided by Schaffner. Drawing on Kuhn’s notion that scientific practices take place in the context of a disciplinary matrix, Schaffner suggests that physicians have at their disposal a matrix of theoretical knowledge consisting of a ‘series of overlapping interlevel temporal models’ [20, p. 68]. He writes:

Clinicians bring to the examination of individual patients a repository of classificatory or nosological generalizations, as well as a grounding in the basic sciences of biochemistry, histology, physiology, and the pathological variants of the ‘normal’ or ‘healthy’ processes. A theory in pathology can be construed as a family of models, each with ‘something wrong’ with the ‘normal’ or ‘healthy’ processes. [20, p. 71]

Schaffner suggests that the pathophysiological mechanisms in individual cases can be understood through application of the theoretical knowledge of the processes represented by these models. He argues that this does not involve the subsumption under universal laws as per Hempel’s covering law account of explanation, but rather, it involves a sort of qualitative comparison which he calls ‘analogical extension of biological knowledge’ [20, p. 68]. The reason for this qualitative comparison is the variability between individuals. As noted in the third section above, individual patients are not tokens of a homogeneous type; they are unique particulars whose histories are influenced by various contingencies. Given this variability, Schaffner argues that the theoretical representations of pathophysiological mechanisms are idealisations:

Such a set of overlapping or ‘smeared out’ models is then juxtaposed, often in a fairly loose way, with an overlapping or ‘smeared out’ set of patient exemplars. This dual ‘smearedness’—one being in the basic biological models and the other in the patient population—typically requires that the clinician work extensively with analogical reasoning and with qualitative and at best *comparative* connecting pathophysiological principles. [20, p. 71]

In other words, the pathophysiological mechanisms represented by the theoretical models at best map partially onto the processes going on in individual cases.

However, as noted in the introductory section, Schaffner’s account is presented as a general account of how theoretical knowledge is applied to cases in the biomedical sciences, not specifically an account of the explanatory functions served by diagnoses. As such, he does not explicitly make clear the particular role that making a diagnosis has in relation to the theoretical knowledge represented by the above mentioned models. It is not clear, for instance, whether he conceives a given diagnosis, such as heart failure, as corresponding to a particular model, a particular node or region in a model, or a process involving multiple models.

When viewed in light of my above analysis of the respective contributions of causal claims and mechanistic causal knowledge, however, the relation between a clinical diagnosis and Schaffner’s matrix of theoretical knowledge is made clear. The contribution of the diagnosis is the identification of the actual cause *C* of the patient data *E*, such as the diagnosis of heart failure identifying the failure of the heart to pump sufficiently as the cause of the patient’s leg oedema. While this description of *C* does not explicitly contain information about the mechanisms by which leg oedema is produced, it is nonetheless implicitly contextualised within a broader matrix of theoretical knowledge consisting of overlapping models of pathophysiological mechanisms. The contribution of this matrix of theoretical knowledge, then, is to provide the background understanding of the mechanisms that make the link between *C* and *E* intelligible. The upshot, then, is that the diagnosis explicitly identifies a pathology whose causal connection with the patient data is made intelligible in virtue of its being contextualised within a theoretical framework of mechanistic models.

It is worth mentioning three additional points to further clarify the relation between a diagnosis and the theoretical models of pathophysiological mechanisms. First, the mechanisms linking a given diagnosis and the patient data may cross a number of these overlapping models. It is usually the case that a disease has sequelae that affect multiple organ systems and span multiple levels. For example, while cystic fibrosis is, in essence, an abnormality of the CFTR ion transport system at the molecular level, it produces histological abnormalities of the mucosal epithelium, which in turn result in anatomical and physiological abnormalities of the gastrointestinal, respiratory, and reproductive systems [23, 24]. Understanding these mechanisms, then, often requires us to invoke models at different levels and of different organ systems. In the case of cystic fibrosis, we need to invoke models of ion transport across the cell membrane, mucous stasis in the airways and pancreatic ducts, chronic inflammation, and so forth.

Second, Schaffner describes the theoretical models each as representing ‘“something wrong” with the “normal” or “healthy” processes’ [20, p. 71], but I suggest that this is not the only way of characterising pathophysiological mechanisms. A recent analysis by Nervi suggests that the theoretical understanding of how *C* and *E* are linked can consist of knowledge about mechanism malfunction, knowledge about pathological mechanisms, or a combination of both [43]. The mechanism malfunction conception involves laying out the details of a normal physiological mechanism and depicting the pathology as an impairment of this normal mechanism. This conception aligns with the theoretical knowledge of ‘pathological variants of the “normal” or “healthy” processes’ described by Schaffner [20, p. 71]. For example, the mechanism of a cardiovascular problem can be explicated by laying out the physiological sequence of events that normally occur in a healthy circulatory system and showing how this sequence is interrupted [43, p. 217]. By contrast, the pathological mechanisms conception lays out the details of the pathological sequence of events without explicit reference to normal physiology. Although background knowledge of normal physiology is presupposed, the emphasis is on the progression of pathological processes. For example, the mechanism of diabetes insipidus can be characterised as decreased production of or sensitivity to the antidiuretic hormone, lack of permeability of cells of the distal nephron, polyuria, dehydration, hypovolaemic shock, and cardiac arrest [43, p. 219].

Third, while I think Schaffner is correct to claim that the theoretical models of pathophysiological mechanisms only partially fit the goings on in actual cases because of the variability across individuals [20], I argue that the diagnosis itself can still be considered a repeatable type, as suggested in the second section above. This is because it is often, though by no means always, the case that a diagnosis is explicitly defined by some essential feature that is necessary for a case to qualify as an instance of that diagnosis. As such, every case of that diagnosis must instantiate that feature. A previously mentioned example from Simon is that of cystic fibrosis, which is explicitly defined by the essential feature of an abnormal CFTR ion transport system, such that ‘regardless of the reason a patient had problems with the CFTR pump system we would consider him to have cystic fibrosis’ [24, p. 361] and that a person who does not have an abnormal CFTR does not, by definition, have cystic fibrosis. Similarly, heart failure is defined by the essential feature of the failure of the heart to pump blood at a rate adequate for satisfying the requirements of the tissues such that only and all patients with heart failure instantiate this feature, despite any variability with respect to their symptoms, signs, and other physiological parameters. Hence, while different cases may deviate from the theoretical models of pathophysiological mechanisms in varying respects and to different degrees, some diagnoses *qua* generalised categories can be taken to pick out certain repeatable processes embedded within the theoretical framework that are conserved across cases.

To put some of the above considerations into context, consider a mechanistic account of how heart failure produces leg oedema from *Davidson’s Principles and Practice of Medicine*:

In patients without valvular disease, the primary abnormality is impairment of ventricular function leading to a fall in cardiac output. This activates neurohumoral mechanisms that in normal physiological circumstances would support cardiac function, but in the setting of impaired ventricular function can lead to a deleterious increase in both afterload and preload…. Stimulation of the renin-angiotensin-aldosterone system leads to vasoconstriction, salt and water retention, and sympathetic nervous system activation. This is mediated by angiotensin II, a potent constrictor of arterioles in both the kidney and the systemic circulation…. Salt and water retention is promoted by the release of aldosterone, endothelin-1 (a potent vasoconstrictor peptide with marked effects on the renal vasculature) and, in severe heart failure, antidiuretic hormone (ADH)…. The onset of pulmonary and peripheral oedema is due to high atrial pressures compounded by salt and water retention caused by impaired renal perfusion and secondary hyperaldosteronism. [45, p. 544]

The above account demonstrates some of the above mentioned features of how theoretical models of pathophysiological mechanisms relate to a diagnosis. First, it describes mechanisms occurring in different organ systems and at different levels, including haemodynamic mechanisms concerning the regulation of blood pressure and cardiac output, hormonal mechanisms concerning the stimulation and actions of the renin-angiotensin-aldosterone system, renal mechanisms of salt and water reabsorption, and the hydrostatic mechanisms of oedema formation. This supports the claim that while a diagnosis may explicitly refer to a pathological process in a particular organ system, understanding the mechanisms by which this produces the patient data may require us to invoke models of several other systems.

Second, in keeping with Nervi’s discussion of the different ways mechanisms can be characterised in medicine, this account includes both information about mechanism malfunction and information about pathological mechanisms. Parts of it characterise the leg oedema resulting from heart failure as being due to interruptions of normal physiological mechanisms, including the impairment of ventricular function. Other parts of it detail the progression of pathological processes leading from heart failure to leg oedema, including stimulation of the renin-angiotensin-aldosterone system, salt and water retention, vasoconstriction, and raised atrial pressure.

Third, in keeping with the notion presented in the fourth section above that the knowledge of the mechanisms by which a pathology produces patient data is useful for the therapeutic aims of clinical medicine, the above account of heart failure identifies potential targets for treatment interventions. For example, stimulation of the renin-angiotensin-aldosterone system can be targeted by angiotensin-converting-enzyme inhibitors, sympathetic nervous system activation can be targeted by β-adrenoceptor antagonists, and salt and water retention can be targeted by loop diuretics. So, while the diagnosis of heart failure tells us what is causing the patient’s leg oedema, the importance of the theoretical understanding of the mechanisms by which it produces the leg oedema is that it indicates where and how to intervene.

## Conclusion

This article has sought to clarify how diagnoses in clinical medicine provide explanations of patient data. Although philosophers have written much about related topics, this particular question has hitherto been underexplored. One of the contributions of this article has been to bring together a range of literature on a number of themes in the philosophy of science and philosophy of medicine—including models of scientific explanation [22], actual causation [21], mechanisms [23], disease ontology [24], and analogical reasoning [20]—in order to arrive at a comprehensive analysis of how diagnoses explain clinical data, positioned within the broader context of these contemporary themes. I have argued that the covering law account is inadequate as a general account of diagnostic explanation, even if the general regularities appealed to are causal regularities, and I endorsed Benzi’s proposal that diagnostic explanation needs to be conceived of as the explanation of particulars based on the notion of actual causation [21]. That is to say, a diagnosis identifies pathology *C* as the actual cause of the patient data *E* in the particular case. However, this simple causal claim is of limited explanatory value without some understanding of the mechanisms by which *C* produces *E*. Drawing on and bringing together Simon’s work on disease ontology [24] and Schaffner’s work on analogical reasoning from theoretical models [20], I argued that this mechanistic knowledge is not explicitly contained in the diagnosis itself, but rather, it comes from the broader theoretical framework within which the causal knowledge provided by the diagnosis is implicitly embedded.
